# 210. Implementation of a Pharmacist Driven Beta-lactam Therapeutic Drug Monitoring Program with Daily Result Reporting: Initial Experiences and Challenges Faced

**DOI:** 10.1093/ofid/ofae631.068

**Published:** 2025-01-29

**Authors:** Samantha Pan, Erin Weslander, Sarah Fierak, Shannon Galvin, Nathaniel J Rhodes

**Affiliations:** Northwestern Memorial Hospital, Chicago, IL; Northwestern Memorial Hospital, Chicago, IL; Northwestern Memorial Hospital, Chicago, IL; Northwestern University Feinberg School of Medicine, Chicago, Illinois; Midwestern University, Downers Grove, IL

## Abstract

**Background:**

Beta-lactam (BL) antibiotics are a cornerstone of treatment for patients with serious infections. Individualized BL dosing using therapeutic drug monitoring (TDM) is an emerging approach to address variability in BL exposure that adversely impacts PK/PD; however, challenges can emerge when implementing TDM programs in the real-world. We sought to characterize the implementation of a BL TDM program by describing initial successes and challenges.

**Figure:**
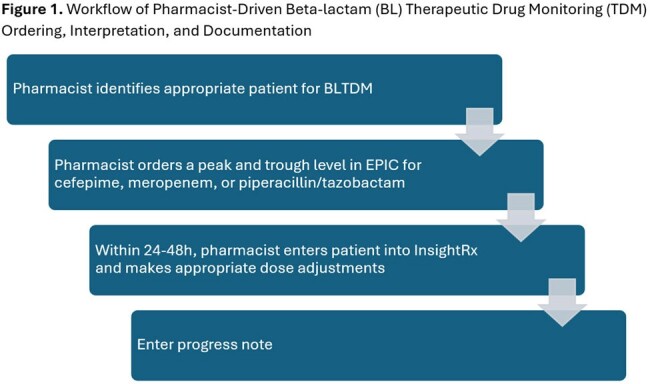

**Methods:**

In September 2023, we implemented a front-line pharmacist driven BL TDM clinical protocol (Figure 1) with daily result reporting for the following BLs: cefepime (FEP), meropenem (MEM), and piperacillin-tazobactam (TZP). Patients with FEP, MEM, and TZP plasma TDM data analyzed between September 26^th^, 2023 and March 31^st^, 2024 were included. TDM results were interpreted using Bayesian analysis (InsightRX). For each TDM event, we characterized ordering provider specialty, whether or not TDM was performed per protocol, whether or not changes were made in response to TDM results, Bayesian model fitness, treatment indication, culture information, and PK/PD target attainment [including predicted steady state trough concentration (Ctrss) and *f*T_> 1xMIC_ and *f*T_> 4xMIC_].

**Figure:**
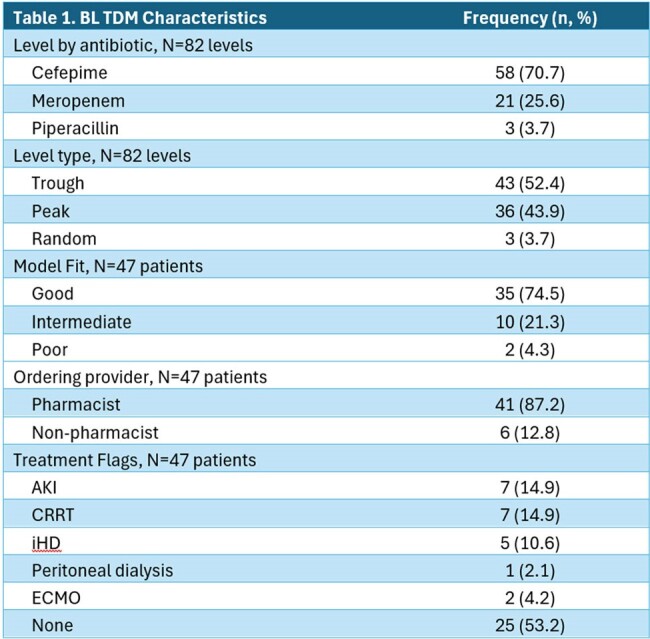

**Results:**

Eighty-two BL TDM levels were obtained in 47 patients. FEP was most frequently monitored (70.7%). Model fit was good or intermediate in > 95% of cases (Table 1). The most common treatment indications were HAP/VAP (n=12, 25.5%), surgical prophylaxis (n=6, 12.8%), bone/joint (n=6, 12.8%), and CNS infection (n=6, 12.8%) (Table 2). Dose adjustments were made in 34% (n=16) of patients. In 57.4% of patients the level order or dose-adjustment deviated from recommended protocols. Targets of 100% *f*T_> MIC_ and 100% *f*T_> 4xMIC_ were achieved in 91.5% and 61.7% of patients, respectively (Table 3). Ctrss was above goal in 21% of patients.

**Figure:**
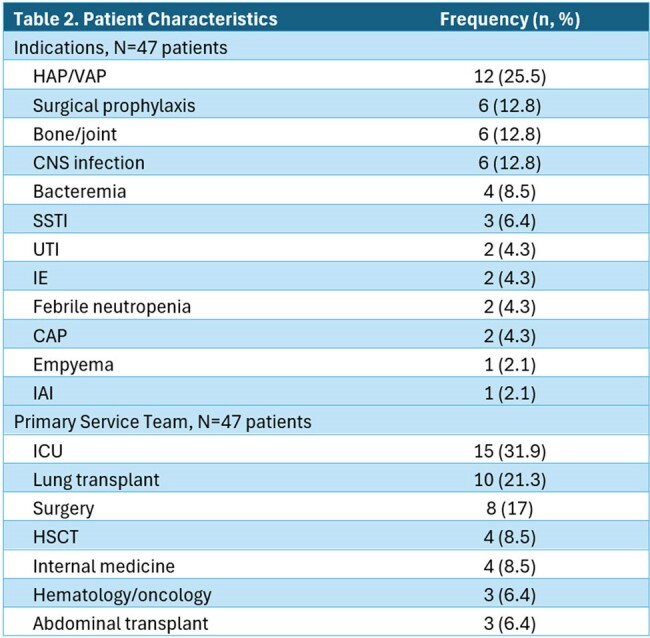

**Conclusion:**

We found that > 90% of patients undergoing BL TDM achieved 100% *f*T_> MIC_, whereas nearly 40% failed to achieve 100% *f*T_> 4xMIC_ at baseline, and > 20% had a Ctrss exceeding safety goals. BL TDM resulted in dose or agent change over 60% of the time, but protocol deviations in dose adjustment were common. The frequency of interventions observed underscores the potential clinical impact of BL TDM on a broader scale when daily reporting is available.

**Figure:**
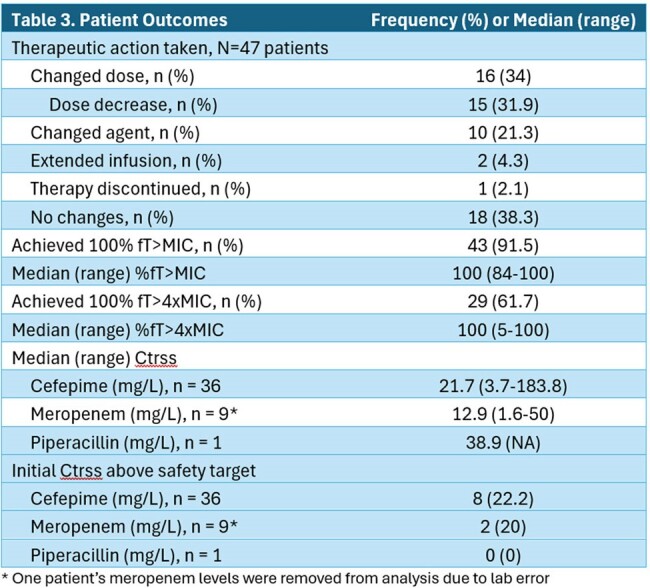

**Disclosures:**

**Nathaniel J. Rhodes, PharmD MS**, Apothecademy, LLC: Advisor/Consultant

